# Supplementary material for: Structure–Tissue Exposure/Selectivity Relationship (STR) on Carbamates of Cannabidiol
Source: Int J Mol Sci. 2024 Nov 5;25(22):11888. doi: 10.3390/ijms252211888 (PMC11593952; doi:10.3390/ijms252211888)
Supplement: Supplementary file 1 [file ijms-25-11888-s001.zip › ijms-3297260-supplementary.pdf]

Supplemental Information

Structure–Tissue Exposure/Selectivity Relationship (STR) on Carbamates of Cannabidiol

Sheng Wang, Jian-Guo Yang, Kuanrong Rong, Huan-Huan Li, Chengyao Wu and Wenjian Tang

Table of contents

1. Preparation of stock solution, standard, and quality control samples........................4
2. Method Validation........................4
2.1 Selectivity........................5
2.2 Linearity and lower limit of quantification........................5
2.3 Precision and accuracy........................5
2.4 Extraction recovery and matrix effect........................5
2.5 Stability........................6
3. Method optimization........................6
3.1 Optimization of liquid chromatography........................6
3.2 Optimization of sample preparation procedures........................7
4. Method validation........................11
4.1 Selectivity........................11
4.2 Linearity and lower limit of quantification........................12
4.3 Precision and accuracy........................13
4.4 Extraction recovery and matrix effect........................15
4.5 Stability........................16
5. ADMET and physicochemical properties prediction of CBD carbamates........................18

Table and figure of Contents for Supporting Information

Supporting Figures and Tables

Fig. S1. Optimization of plasma treatment methods. Comparison of protein precipitation method, liquid-liquid extraction method and solid-phase extraction methods. (A) 1: PP (MeOH), 2: PP (ACN), 3: PP and enrichment (MeOH), 4: PP and enrichment (ACN), 5: LLE (MTBE), 6: LLE (ACN: MTBE = 1: 4), 7: SPE; (B) Comparison of different extractants; (C) Comparison of different extraction time; (D) Comparison of MTBE with different volumes; (E) Comparison of different reconstitution solvent.

Fig. S2. Specific chromatograms of each component in rat plasma. (A) Blank plasma; (B) Blank plasma with five compounds at the Lower Limit of Quantification and internal standard; (C) Blank plasma with six compounds at the MOQ; (D) Plasma of one hour after oral administration of each drug to rats.

Table S1 Parameters optimized with the developed method by UPLC-HRMS.

Table S2 Standard curve line and Detection Limits of the detection of CBD (**L0**) and **L1**–**L4**.

Table S3 Intra-day and inter-day precision and accuracy of each analyte (n = 5).

Table S4 Extraction recovery rate and matrix effect of the detection of each analyte (n = 5).

Table S5 The stability of each component under different storage conditions

Table S6 Descriptors collected from Mat-ADMET.

1. Preparation of stock solution, standard, and quality control samples

The stock solution (1 mg/mL of methanol) of analytes (CBD, **L1–L4**) was diluted in methanol to prepare 6, 15, 30, 75, 150, 300, 600 ng/mL mixed reference working solution. The low QC (LQC), medium QC (MQC) and high QC (HQC) working solution were prepared with the same spiking procedure at concentrations of 13.5, 135 and 315 ng/mL for analytes. The internal standard (IS, **L5**, CBD 4-bromobenzyl (methyl)carbamate) was dissolved in methanol, and diluted in methanol to prepare IS working solution (300 ng/mL for plasma, and 1000 ng/mL for tissue). All standard solutions were stored at –20 °C and plasma samples were stored at –80 °C until analysis.

For plasma sample, the mixed working solutions were further diluted in blank plasma to prepare spiked serum calibrants (n = 7), **L0–L4** at 2, 5, 10, 25, 50, 100, and 200 ng/mL; Quality control (QC) samples were similarly prepared by spiking blank plasma with the targeted compounds at low LQC, MQC, and HQC concentrations, which were respectively 4.5, 45 and 105 ng/mL for **L0–L4**; For tissue sample, the mixed working solutions were further diluted in blank tissue (brain, heart, kidney, liver, lung or spleen) to prepare spiked tissue calibrants (n = 7), **L0–L4** at 5, 12.5, 25, 62.5, 125, and 250 ng/g. The plasma and tissue sample proceeded as sample pretreatment showed in ***Section 2.3*** for injection.

2. Method Validation

The optimized bioanalytical method for CBD (**L0**) and its carbamates (**L1–L4**) was fully validated based on the current guidelines and acceptance criteria recommended by the US Food and Drug Administration (FDA) guidelines (US Food and Drug Administration, Guidance for Industry, Bioanalytical Method Validation, 2019) in terms of selectivity, linearity, sensitivity, precision, accuracy, matrix effect, recovery and stability under different storage conditions.

2.1 Selectivity

The specificity of the method was by comparing samples from 5 “blank” SD rats within/without analytes and IS. If the response of the endogenous interfering substance at the retention time of the analyte is less than 20% area of the lower of quantification (LOQ), and the interfering area at the retention time of internal standard is less than 5% intensity of working internal standard, indicating that the targets and internal standard measurements were not affected by impurities or endogenous compounds.

2.2 Linearity and lower limit of quantification

The linearity of calibration curve was assessed by analyzing seven concentration levels of standard plasma samples and constructed by plotting the peak area ratios (Y) of **L0–L4** to IS against the nominal concentration samples in plasma of compounds (X, ng/mL), using a $1/X^2$ weighted linear least squares regression model. The sensitivity was determined by analyzing six repeated plasma added low quantitative samples. The back calculated concentration of each calibration standard must be within 15% of the nominal value.

2.3 Precision and accuracy

Intra-batch and inter-batch precision and accuracy were measured by analyzing QC samples at three concentration levels (LQC, MQC and HQC) with five determinations in three consecutive validation runs. Intra-batch and inter-batch precision were assessed by One-way analysis of variance (ANOVA). Intra-batch and inter-batch precision are expressed as relative standard deviation (RSD) and shall not exceed 15%.

2.4 Extraction recovery and matrix effect

Under LQC and HQC, the recovery of each analyte is determined by analyzing the repetition of five QC samples, and calculated by the ratio of peak area of spiked samples extracted under corresponding concentration to that of non-extracted standards. Five different batches of blank plasma were analyzed and standard

solutions were added at LQC and HQC concentrations to evaluate the matrix effect. Matrix effect is expressed as the ratio of the peak area of blank plasma sample extracted after standard addition with pure standard solution to the peak area of corresponding concentration of pure standard solution. The IS normalized matrix factor is used to express the ratio of the matrix factor of each analyte to the matrix factor of IS to evaluate the effect of matrix on ionization.

2.5 Stability

The stability of each analyte in plasma was studied under LQC and HQC conditions at room temperature, three freeze-thaw (–20 °C) cycles and long-term (–20 °C) storage for 30 days.

3. Method optimization

3.1 Optimization of liquid chromatography

In this study, the chromatographic performance of different chromatographic columns was evaluated, including Shimadzu-pack GIST column (50 mm × 2.1 mm, 2 μm), Waters Acquity UPLC HSS T3 (2.1 mm × 100 mm, 1.8 μm) and Agilent Zorbax Eclipse Plus C_{18} (2.1 mm× 50 mm, 1.8 μm). Because of the low polarity of **L0–L4**, the analytes exhibites a longer retention at Waters Acquity UPLC HSS T3 (2.1 mm × 100 mm, 1.8 μm) column. Compared with the Agilent Zorbax Eclipse Plus C_{18} (2.1 mm× 50 mm, 1.8 μm) column, the Shimadzu-pack GIST column (50 mm × 2.1 mm, 2 μm) column provided a symmetrical peak shape and the most efficient retention of the analyte targets. Therefore, a Shimadzu-pack GIST column was used for analyses.

The **L4** and **L5** have very similar chemical structure, the **L4** show some interference at the PRM channel of the **L5** (IS), this effect aggravates with increasing concentration of **L4**, the results were unacceptable when **L4** at a high concentration. So the chromatographic separation of **L4** and **L5** was the crucial factor in accurate quantification in this study. We carefully optimized elution gradient, and the **L4** and **L5** have reach the completely separation on the optimal liquid chromatographic

condition show in ***Section 2.4***.

Table S1

Parameters optimized with the developed method by UPLC-HRMS.

Compd.	Mol. Wt.	Molecular formula	Precursor ion (*m/z*)	CE (*eV*)	Product ion (*m/z*)	RT/min
CBD (**L0**)	314.46	$C_{21}H_{30}O_2$	315.23186	20	193.12215	3.70
**L1**	371.51	$C_{23}H_{33}NO_3$	372.25332	15	315.23117	3.70
**L2**	399.57	$C_{25}H_{37}NO_3$	400.28462	13	266.17456	4.77
**L3**	411.58	$C_{26}H_{37}NO_3$	412.28462	13	278.17453	4.75
**L4**	496.08	$C_{30}H_{38}ClNO_3$	496.26130	13	297.22083	6.21
**L5 (IS)**	540.54	$C_{30}H_{38}BrNO_3$	540.21078	13	297.22134	6.47

3.2 Optimization of sample preparation procedures

Sample preparation is a key factor in the development of bio-analytical methods to achieve maximum extraction recovery, minimum matrix effect and easiest sample preparation procedures. We compared three plasma pretreatment methods: protein precipitation, liquid-liquid extraction and solid-phase extraction.

For protein precipitation test, we set four groups to compare the efficiency on MQC samples, group 1 using 180 μL MeOH, group 2 using 120 μL ACN, group 3 using 180 μL MeOH and dried under nitrogen for enrichment, group 4 using 120 μL ACN and dried under nitrogen for enrichment. For liquid-liquid extraction, MTBE (group 5) and ACN: MTBE = 1: 4 (group 6) were selected for extraction reagent. The solid phase extraction (SPE, group 7) was also tested on Waters Oasis HLB cartridges (3 cc/60 mg; Waters). The results are shown in Fig. S1A.

As for protein precipitation method, the response value of group 1 was higher than that of group 2 for the five compounds, the MeOH show a better result than ACN. Compared with protein precipitation group, the SPE show better results for **L4**, while the **L0-L3** have no obviously improvement. The liquid-liquid extraction, especially the MTBE group show the best results for all compound, Finally, liquid-liquid extraction was selected and get further optimization.

In liquid-liquid extraction optimization, MTBE, ethyl acetate (EAC), chloroform ($CHCl_3$), and $CHCl_3$: MTBE (1: 2) were compared, the data are exhibited in Fig. S1B. Among the four groups, chloroform had the lowest extraction effiency, and MTBE had the highest response in **L0**, **L1** and **L4**. For **L2**, all group performed nearly similar response. For **L3**, except for chloroform group, there was little difference in the response of the other three group, MTBE was selected as extractant. In order to get better sensitivity in liquid-liquid extraction, different MTBE volumes (400, 500, 600, and 700 μL), extraction times (1, 2, 5, and 10 min) and composition of the reconstitution solvent were further compared, the results were exhibited in Fig. S1C–S1E. Finally, for the plasma sample preparation, we choose 400 μL MTBE and set the extraction time at 10 min, 70% methanol was optimized and chose as the reconstitution solvent.

During the experiment, the linearity of the analytes was always not ideal, we considered that the compounds in the plasma were oxidized during the preparation process because of the phenolic hydroxyl group in the cannabidiol and its carbamates. We added extra volume 0.1% VC aqueous solution during the sample preparation, the results were shown in Figure S1F. The results confirmed our hypothesis, the VC aqueous solution promote the stability of **L0–L5**, especially for **L4**. The optimal sample preparation procedures show in ***Section 2.3***.

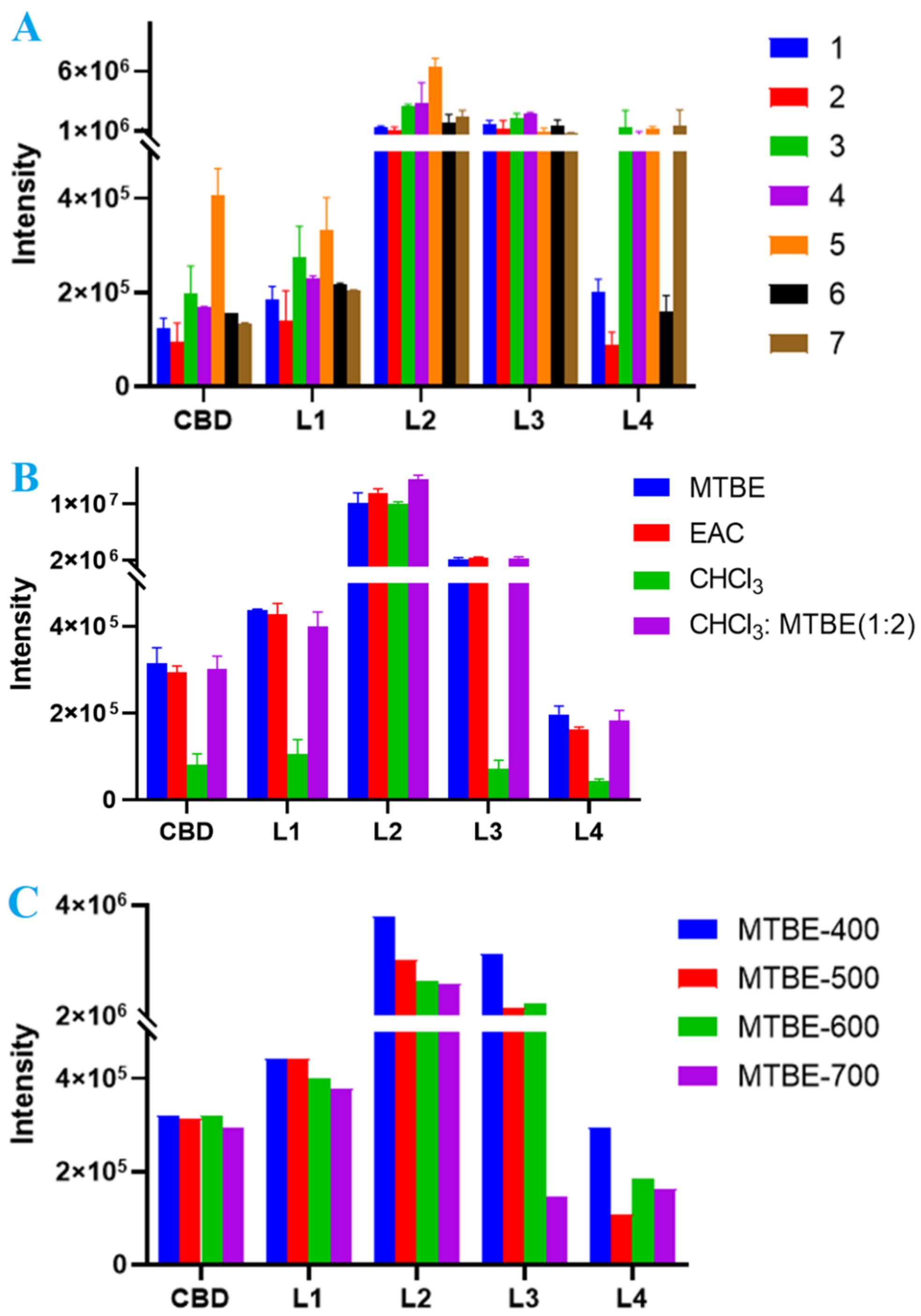

A
6×10^6
1×10^6
4×10^5
2×10^5
0
Intensity
CBD
L1
L2
L3
L4
1
2
3
4
5
6
7
B
1×10^7
2×10^6
4×10^5
2×10^5
0
Intensity
CBD
L1
L2
L3
L4
MTBE
EAC
CHCl3
CHCl3: MTBE(1:2)
C
4×10^6
2×10^6
4×10^5
2×10^5
0
Intensity
CBD
L1
L2
L3
L4
MTBE-400
MTBE-500
MTBE-600
MTBE-700

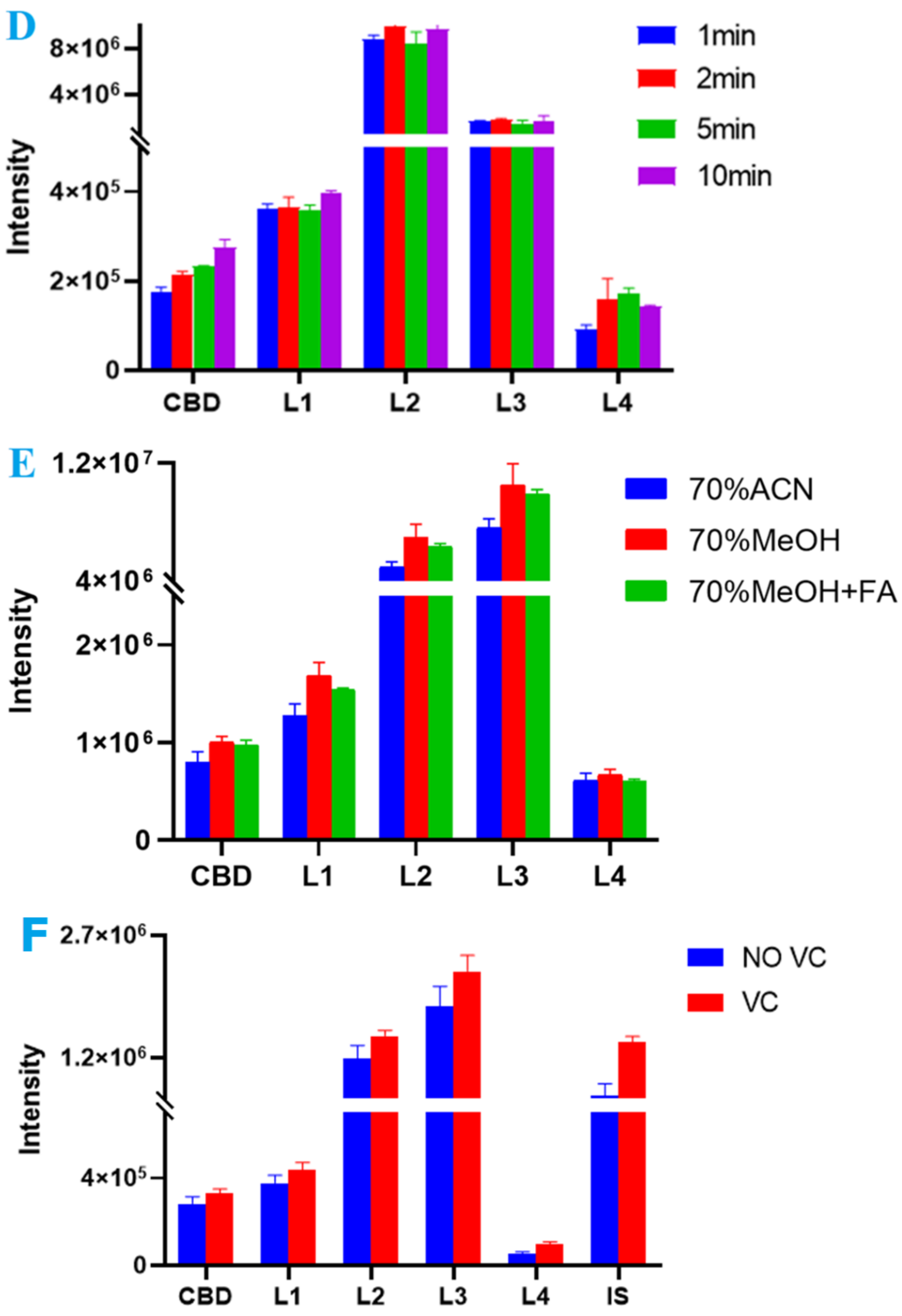

Fig. S1. Optimization of plasma treatment methods. Comparison of protein precipitation method, liquid-liquid extraction method and solid-phase extraction methods. (A) 1: PP (MeOH), 2: PP (ACN), 3: PP and enrichment (MeOH), 4: PP and enrichment (ACN), 5: LLE (MTBE), 6: LLE (ACN: MTBE = 1: 4), 7: SPE; (B) Comparison of different extractants; (C) Comparison of different extraction time; (D) Comparison of MTBE with different volumes; (E) Comparison of different reconstitution solvent; (F) Comparison of aqueous solution containing 0.1% VC.

4. Method validation

4.1 Selectivity

Fig. S2 is a typical chromatogram of CBD and its carbamates in blank plasma and corresponding samples. No obvious interference peak was observed in the retention time of analytes and ISS. No significant carrying peaks were observed in analytes and ISS. The retention times of CBD, **L1**, **L2**, **L3**, **L4** and IS were 3.70, 3.70, 4.77, 4.75, 6.21 and 6.47 minutes respectively.

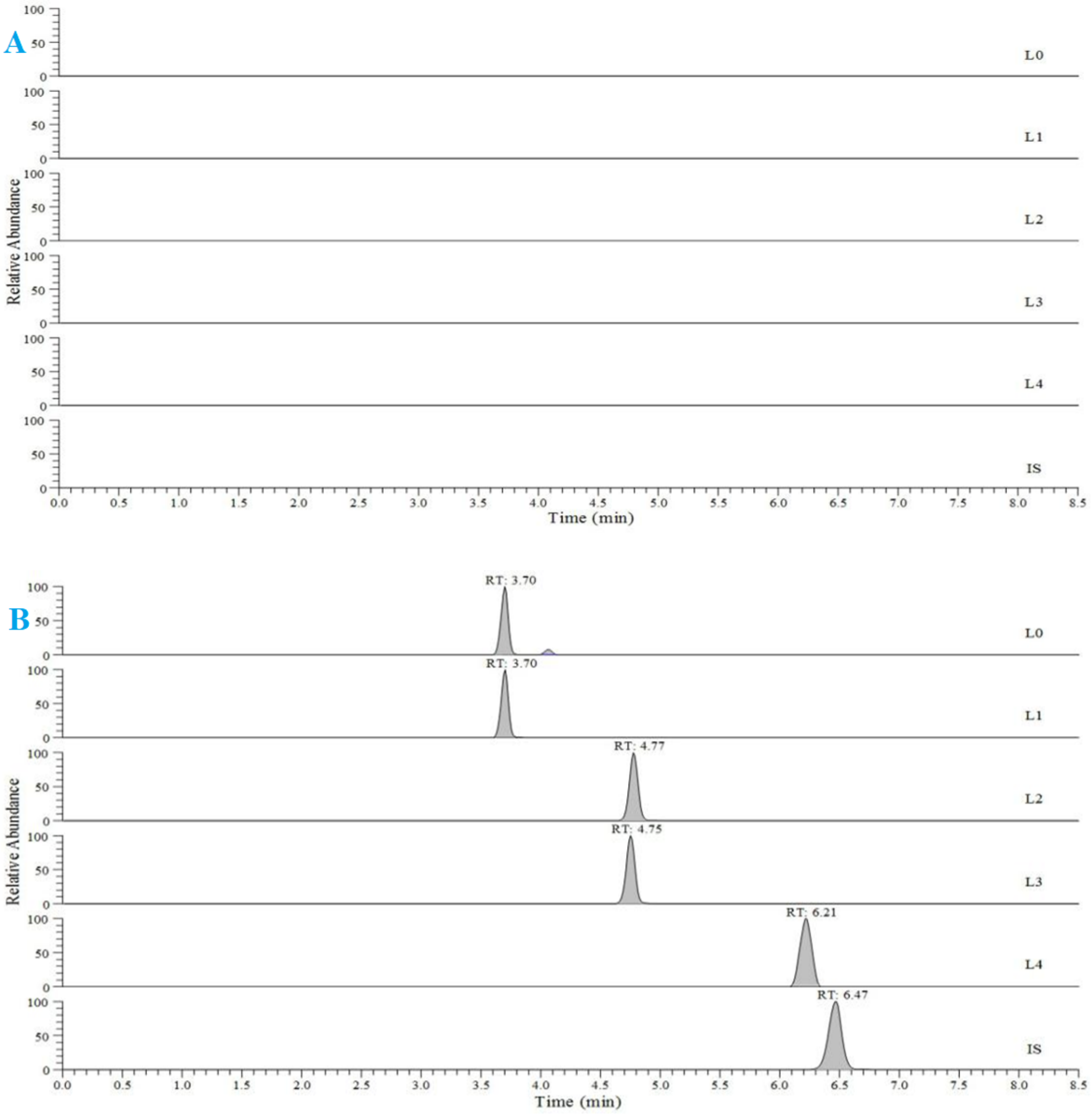

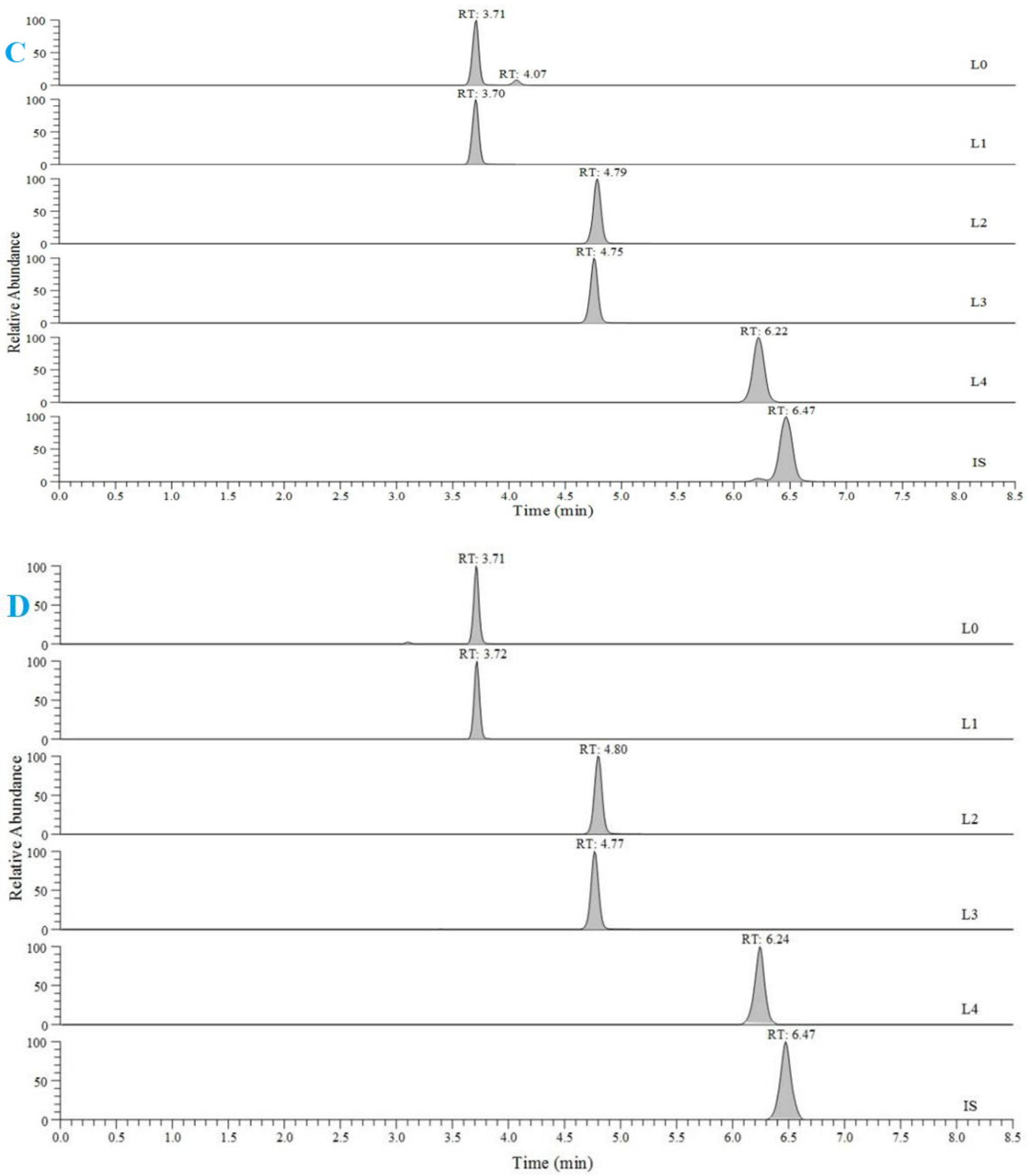

Fig. S2. Specific chromatograms of each component in rat plasma. (A) Blank plasma; (B) Blank plasma with five compounds at the Lower Limit of Quantification and internal standard; (C) Blank plasma with six compounds at the MOQ; (D) Plasma of one hour after oral administration of each drug to rats.

4.2 Linearity and lower limit of quantification

The typical calibration curves of the five compounds in the range of 2–200 ng/mL have good linearity and the correlation coefficient is satisfactory (R^2 > 0.9931). The LLOQ of CBD and carbamates in the plasma was 2 ng/mL, indicating that the

established method has the excellent sensitivity. The sensitivity of the method was adequate for generating plasma concentration time profiles of CBD and its derivatives after oral to rats (Table S2).

4.3 Precision and accuracy

The spiked quality control samples prepared and analyzed as described above were used to evaluate the intra batch precision, inter-batch precision and accuracy at low, medium and high concentrations. The calculation data are shown in Table S3. The precision was below 15% at all inter assay and inter assay levels. The established method has good intra batch and inter batch precision and accuracy. The accuracy value is in the range of 92.56%–110.74%, which is within the acceptable range of this parameter.

Table S2

Standard curve line and Detection Limits of the detection of CBD (**L0**) and **L1**–**L4**.

Analyte	Linear range (ng/mL)	Linear regression equation	Correlation coefficient (r)	LOQ (ng/mL)	RSD of LOQ (n = 6, %)
CBD (**L0**)	2-200	Y = -0.0134+0.0583X	0.998	2	8.42
**L1**	2-200	Y = -0.0228+0.07957X	0.998	2	5.39
**L2**	2-200	Y = 0.0795+0.1680X	0.997	2	14.21
**L3**	2-200	Y = 0.0555+0.2546X	0.998	2	12.82
**L4**	2-200	Y = -0.0028+0.0160X	0.999	2	12.38

Table S3

Intra-day and inter-day precision and accuracy of each analyte (n = 5).

Analyte	Spiked conc. (ng/mL)	Inter-batch RSD (%)	Intra-batch RSD (%)	Inter-accuracy (%)	Inter-accuracy RSD (%)	Intra-accuracy (%)	Intra-accuracy RSD (%)
CBD (**L0**)	4.5	14.53	2.86	100.02	6.10	92.56	3.26
	45	6.61	4.54	102.47	4.89	100.73	7.12
	105	1.69	3.42	101.69	3.23	102.55	4.78
**L1**	4.5	14.46	3.06	99.93	6.16	94.12	3.43

	45	14.94	3.28	97.38	6.41	93.97	3.23
	105	4.61	2.71	98.48	3.05	97.09	3.94
**L2**	4.5	5.01	1.91	108.98	2.59	110.74	1.46
	45	4.77	4.84	103.17	4.83	101.31	6.04
	105	1.51	4.96	100.90	4.63	100.69	4.51
**L3**	4.5	2.35	5.26	107.96	4.95	107.57	7.10
	45	9.73	3.48	107.12	4.89	103.64	5.69
	105	5.67	4.93	103.69	5.04	105.84	5.98
**L4**	4.5	3.13	3.97	107.11	3.86	105.53	5.89
	45	8.11	5.88	100.67	6.24	98.45	6.28
	105	12.70	7.42	99.84	8.38	102.45	4.61

4.4 Extraction recovery and matrix effect

No obvious matrix effect was observed in the quality control samples spiked with all analytes at low and high concentrations, and the corresponding value was within the specified range. The results showed that the extraction was successful, and no obvious matrix effect was observed (Table S4).

Table S4

Extraction recovery rate and matrix effect of the detection of each analyte (n = 5).

Analyte	Spiked conc. (ng/mL)	MF_{Sample}% (mean, RSD)	MF_{IS}% (mean, RSD)	Extraction recovery (%) (mean, RSD)	Relative recovery (%) (mean, RSD)
CBD **(L0)**	4.5	(90.95, 3.59)	(97.01, 7.41)	(67.25, 4.69)	(86.15, 8.38)
	105	(93.60, 2.91)	(97.86, 1.49)	(74.82, 8.71)	(81.92, 6.97)
**L1**	4.5	(93.72, 4.93)	(101.01, 4.63)	(64.16, 9.52)	(83.09, 12.71)
	105	(92.70, 2.86)	(105.63, 4.74)	(77.64, 5.8)	(80.26, 7.64)
**L2**	4.5	(94.94, 7.54)	(98.15, 8.09)	(75.41, 10.93)	(93.41, 11.55)
	105	(99.78, 6.07)	(104.26, 3.60)	(93.40, 6.61)	(102.23, 5.42)
**L3**	4.5	(94.26, 5.36)	(97.51, 8.55)	(70.30, 12.85)	(87.26, 14.22)
	105	(100.27, 3.81)	(106.31, 0.06)	(88.06, 5.99)	(97.75, 7.94)
**L4**	4.5	(91.56, 14.08)	(93.55, 14.84)	(104.48, 11.75)	(129.41, 11.79)
	105	(91.66, 4.43)	(93.66, 1.60)	(112.58, 5.57)	(123.35, 5.97)

4.5 Stability

Table S5 summarized the results of stability test. The results showed that the five compounds in rat plasma samples were stored at room

temperature for 12 hours under different storage conditions, the three freeze-thaw cycles were stable, and the variation range of concentration was within 15% of the actual value. Under the condition of long-term (–20°C) storage for 30 days, the compound is unstable and **L4** is lost more.

Table S5

The stability of each component under different storage conditions

Analyte	Spiked conc. (ng/mL)	Repeated freeze-thaw stability (%, mean, RSD) (n = 12)	Long-term frozen storage stability at -20°C (%, mean, RSD) (n = 3)	Short-term room temperature stability at 25°C, 2h (%, mean, RSD) (n = 3)
CBD (**L0**)	4.5	(109.32, 6.03)	(106.17, 6.95)	(99.49, 4.45)
	105	(97.82, 4.72)	(96.89, 0.59)	(102.57, 10.57)
**L1**	4.5	(106.42, 5.38)	(112.88, 3.35)	(101.41, 6.13)
	105	(97.22, 3.09)	(91.91, 3.61)	(100.35, 7.68)
**L2**	4.5	(102.76, 5.50)	(94.88, 8.59)	(95.46, 4.70)
	105	(95.72, 7.62)	(87.07, 5.30)	(95.67, 7.89)
**L3**	4.5	(100.54, 4.81)	(99.31, 7.37)	(93.38, 6.43)
	105	(96.71, 5.93)	(88.63, 4.19)	(100.70, 9.21)
**L4**	4.5	(97.49, 3.27)	(68.90, 22.89)	(96.04, 3.35)
	105	(94.95, 4.59)	(74.43, 5.85)	(100.68, 9.61)

5. ADMET and physicochemical properties prediction of CBD carbamates

Table S6

Descriptors collected from Mat-ADMET.

name	CBD **(L0)**	**L1**	**L2**	**L3**	**L4**
**Molecular Weight**	314.469	371.521	399.575	411.586	496.091
**nHA**	2	3	3	3	3
**nHD**	2	2	1	1	1
**nRot**	6	7	8	7	9
**nAlipRing**	1	1	1	2	1
**nAromRing**	1	1	1	1	2
**nRing**	2	2	2	3	3
**fChar**	0	0	0	0	0
**TPSA**	40.46	58.56	49.77	49.77	49.77
**LogP**	6.455	5.887	6.710	6.913	8.053
**LogD**	4.792	4.111	4.181	4.076	4.817
**LogS**	-4.654	-4.300	-3.840	-4.061	-5.751
**Solubility**	0.01	0.014	0.021	0.017	0.003

**FreeSolv**	-6.347 kcal/mol	-8.884 kcal/mol	-8.792 kcal/mol	-9.512 kcal/mol	-9.006 kcal/mol
**QED**	0.511	0.466	0.396	0.399	0.280
**SAscore**	3.461	3.589	3.645	3.522	3.551
**Fsp3**	0.524	0.522	0.560	0.577	0.433
**NPscore**	1.985	1.788	1.367	1.263	0.783
**MCF**	0	0	0	0	0
**PAINS**	0	0	0	0	0
**F30%**	0.375	0.614	0.466	0.484	0.683
**F50%**	0.414	0.418	0.409	0.443	0.492
**Pgp-inhibitor**	0.128	0.878	0.102	0.994	1
**Pgp-substrate**	0	0	0.005	0.001	0.003
**HIA**	1	1	1	1	1
**BBB Penetration**	0.915	0.804	0.957	0.921	0.902
**hPPB**	100.00%	100.00%	99.76%	94.14%	100.00%
**VDss (Human)**	2.047 L/kg	1.634 L/kg	1.368 L/kg	1.369 L/kg	1.478 L/kg
**VDss (Dog)**	0.916 L/kg	1.051 L/kg	1.080 L/kg	0.973 L/kg	1.200 L/kg
**VDss (Monkey)**	1.405 L/kg	1.226 L/kg	1.375 L/kg	1.367 L/kg	1.556 L/kg

**VDss (Mouse)**	1.655 L/kg	1.319 L/kg	1.347 L/kg	1.557 L/kg	1.542 L/kg
**VDss (Rat)**	2.503 L/kg	1.890 L/kg	1.774 L/kg	2.042 L/kg	1.905 L/kg
**CYP1A2 inhibitor**	0.200	0.130	0.023	0.054	0.463
**CYP2C19 inhibitor**	0.993	1	1	1	1
**CYP2C9 inhibitor**	0.001	0.005	0.005	0.570	1
**CYP2D6 inhibitor**	0.637	0.376	0.661	0.334	0.763
**CYP3A4 inhibitor**	0.972	0.940	0.813	0.960	0.910
**CYP2D6 substrate**	0.091	0.171	0.067	0.204	0.061
**CYP3A4 substrate**	0.082	0.142	0.660	0.777	0.827
**CYP2C9 substrate**	1	1	1	1	1
**BCRP inhibitor**	1	1	1	1	1
**T1/2**	8.091 hour(s)	6.094 hour(s)	6.015 hour(s)	8.595 hour(s)	8.273 hour(s)
**hERG Blocker**	0.001	0.009	0.131	0.538	0.361
**Eye-Corrosion**	0	0	0	0	0
**AMES**	0.001	0.007	0.061	0.023	0.048
**Anticomensal Effect**	0.594	0.463	0.662	0.213	0.995
**DILI**	0.003	0.002	0.01	0.001	0.002

**Skin Reaction**	1	1	1	1	0.999
**Myelo Toxicity**	1	0	1	0	0.213
**Hemolytic Toxicity**	0.370	0.277	0.232	0.265	0.179
**NR-AR**	0	0	0	0	0
**NR-AR-LBD**	0.053	0.017	0.033	0.025	0.041
**NR-AhR**	0.001	0.001	0.003	0.003	0.003
**NR-Aromatase**	0.058	0.060	0.053	0.048	0.037
**NR-ER**	0.113	0.061	0.007	0.015	0.049
**NR-ER-LBD**	0	0	0	0	0
**NR-PPAR-gamma**	0.008	0.01	0	0	0
**SR-ARE**	1	1	0.997	1	1
**SR-ATAD5**	0	0	0	0	0
**SR-HSE**	0.116	0.001	0.003	0.007	0.004
**SR-MMP**	1	0.989	0.908	0.995	0.986
**SR-p53**	0.255	0.232	0.414	0.348	0.159
**Rat acute oral toxicity (LD50)**	319.474	22.061	70.497	80.045	84.020
